# Alcoholic Adulteration

**Published:** 1890-07

**Authors:** S. H. Preston


// HALL'S
### JOURNAL OF HEALTH

TRUTH DEMANDS NO SACRIFICE; ERROR CAN MAKE NONE.

Vol. 37. JULY, 1890. No. 7.

### ALCOHOLIC ADULTERATION.

BY S. H. PRESTON.

Alcohol in America is probably a poison in a more special sense than is popularly supposed. It is a chemically attested fact, but one only affecting those who drink, that almost all the beer made in the United States is little else than vile slops, and nearly all spirituous liquors are intolerable drugged intoxicants. If Congress should ever concern itself with sumptuary measures, it ought to put an embargo on the whole lot. The consumers are mostly people addicted to drink by a habit which senses slight squeamishness on the score of adulteration, and who take their toddy for its stimulating effect, and it matters little what be its chemical constituents or corrosive action.

There are temperance ranters who reason that the more deadly the drink, and the quicker it destroys the drunkard, the better. But those who feel for their unfortunate fellows will never despair of their reformation, and will regard an ounce of prevention better than a pound of kill or cure, as applied to inebriates. The appetite for intoxicating drink is doubtless due in a great degree to the poisonous ingredients in the drink itself, which cause or aggravate the cursed craving for stimulants. And if people are to have gin at all, it is better to give them good gin, or liquor of a quality that will not fire them with a feverish thirst for more.

Aside from the subject of adulterated drink, is this to be thought of by temperance reformers—a good cook will serve better to reclaim a wretch from the ruin of rum than a presbytery of pharisaical parsons—roast beef better than religious rant or reiterated resolutions. The craving for alcoholic stimulants usually comes from a physical system not properly nourished, or one that is overworked. The nations in which drunkenness is most prevalent are those whose people have least to eat, and among classes confined to poor and unwholesome articles of food. We find drunkenness most common in our city slums, where good and nourishing provisions properly prepared are rarely tasted.

The great temptation of alcohol is the speedy effect of it as a stimulant ; and any person can procure all the stimulant required from a proper amount of hearty, invigorating food. The weary workman who slips out of his shop for a ten cent drink of old rye to renew his strength, would do far better to get ten cents worth of soup at a restaurant, and the street laborer who spends a car fare for a glass of beer, would get more comfort and cheer by buying five cents worth of bread and bacon.

A man who can afford to drink can afford to eat ; and if our government will take the matter in hand of improving the quality of cheap food commodities, and the temperance people expend their efforts in putting that food into the drink-diseased stomachs of the poor victims of alcohol, we may hope for a decrease of drunkenness at no distant day.

